# Water Use Efficiency and Tomato Yield Under the Influence of Irrigation Water Quality and Soil Improvers Using a Drip Irrigation System

**DOI:** 10.3390/plants15050734

**Published:** 2026-02-28

**Authors:** Hussein R. Nayyef, Mohammed A. Naser, Flaieh Hammed Kassar, Yahya Jihad Shabeeb, Wisam Bisheer Hasan, Amin Hussain Jabal

**Affiliations:** 1 Department of Tissue Culture and Medicinal Plants Techniques, Shatrah Technical College, Southern Technical University, Basrah 61001, Iraq; hussein.razzaq@stu.edu.iq; 2 Department of Combating Desertification, College of Agriculture, Al-Muthanna University, Al-Samawah 66001, Iraq; flaiehkassar@mu.edu.iq; 3 Department of Soil Science and Water Resources, College of Agriculture, University of Basrah, Basrah 61001, Iraq; yahia.shabib@uobasrah.edu.iq (Y.J.S.); amin.hussain@uobasrah.edu.iq (A.H.J.); 4 Department of Project Management, Administration and Economics, Al Qurna College, University of Basrah, Basrah 61001, Iraq; wisam.hasan@uobasrah.edu.iq

**Keywords:** water use efficiency, water productivity, mean weighted diameter, irrigation water conservation

## Abstract

As a result of Iraq’s scarcity of fresh water, there is a need to find alternative, non-traditional irrigation methods and technologies that would increase water use efficiency and reduce the negative impact of salinity on the tomato crop. The experiment was conducted in the field over two consecutive seasons in heavy soil using a drip irrigation system. The study employed two types of irrigation water with different salinity levels (low, symbolized as q1 = 0.8 ds m^−1^) and high, symbolized as (q2 = 5.8 ds m^−1^), and added in three ways: Q1 (q1), Q2 (q1, q2), and Q3 (q2). Two levels of organic matter (F2 and F3) were also used, along with a control treatment without the addition of F1. The study aimed to evaluate the effect of alternating fresh and saline water on tomato productivity, as well as to determine the impact and effectiveness of organic fertilizer in mitigating the negative effects of saline irrigation water and improving the chemical and physical properties of the soil. Statistical analysis showed that both irrigation water quality and amendments had a significant effect on the studied properties. The study year did not affect the overall characteristics of the study, but only the water conductivity and weighted diameter. The results showed an increase in water use efficiency, with averages of 20.7 and 21.13 kg ha^−1^ mm^−1^. when using fresh water and a high level of organic matter addition, sequentially. The water quality treatment Q2, combined with soil amendment F3, achieved the highest yield compared to the fresh water treatment Q1 combined with a control treatment (F1), reaching 4.321 and 3.993 kg plant^−1^, respectively. This was achieved while conserving fresh water by 50% when using moderately saline drainage water with added amendments, without a significant decrease in tomato yield. Therefore, this study proposes adopting a strategy of using saline water with medium electrical conductivity as a partial alternative to low-salinity water, while incorporating organic amendments to ensure sustainable production in water-scarce regions.

## 1. Introduction

The growing water crisis is a clear and significant threat to progress towards ensuring sustainable development in semi-arid and arid regions, especially in the Middle East. Expectations indicate an increase in food imports as a result of the rapid population growth in these regions. This population increase calls on us to preserve and develop water resources in all their forms, whether non-fresh surface water or groundwater. In addition, the clear expansion in agriculture and industrial sectors has caused significant and increasing pressure on water resources [1]. Agricultural yields are affected by a range of difficulties, including freshwater scarcity and stress caused by desertification and drought [2]. Therefore, there are urgent and necessary requirements and sustainable strategies for water management, especially in countries with arid climates such as Iraq. The average water release rate of the Tigris and Euphrates rivers was (610) cubic meters second^−1^ and (850) cubic meters second^−1^ in 2019, respectively. Meanwhile, the amount of water entering Iraq in October 2025 for the Tigris and Euphrates rivers was (200) cubic meters second^−1^ and (151) cubic meters second^−1^, respectively [3].

Freshwater resources are under increasing pressure as a result of irrigated agriculture. This, in turn, will push agricultural producers to use and adopt modern irrigation technologies that increase crop productivity and reduce the use of irrigation water [4,5]. Most studies confirm that more than 70–80% of water is consumed as a result of irrigated agriculture worldwide, and that both scarcity and shortage bring damage and problems to the soil, represented by its erosion and increased salinity [2,6]. As a result of the unscientific use of irrigation water to water agricultural lands and its scarcity from the source, the amount of water in the Tigris and Euphrates rivers in our country, Iraq, has decreased. The soil environment of agricultural lands has changed as a result of climate change and the desertification that the country is going through, and the scarcity of cultivated areas due to the shortage of fresh water from the source, as well as the decrease in the percentage of rainfall in recent years. Therefore, exploring ways and means to reduce the use of fresh water in irrigating crops and investing in drainage water and lower quality groundwater due to high salinity levels under a freshwater-saving regeneration project can provide a practical basis for the sustainable development of agriculture under the conditions of water-saving regeneration initiatives in agricultural areas in the country [7,8].

Due to frequent extreme weather events, these climate changes have clearly and significantly affected vegetation cover rates and water quantities in terrestrial ecosystems. Water use efficiency (WUE) is defined as the ratio of Gross primary productivity (TPP) to evaporation and transpiration (ET), which represents the rate of irrigation water evaporation per unit of absorbed carbon [5,9]. The WUE is linked to the carbon and water cycle processes in ecosystems and serves as a key indicator for measuring the response of ecosystem outputs to water resources [10]. Therefore, a comprehensive analysis of variations in irrigation water quality, the quantity of water use efficiency improvers, and their driving factors contributes to elucidating the carbon and water cycle processes in ecosystems and their responses to climate change.

With increasing irrigation levels, water use efficiency increases, but it has begun to decrease, and at the highest irrigation level, when using four irrigation levels of 60, 80, 100, and 120% of the reference evaporation, the decrease in efficiency is attributed to the interference of several chemical, physiological, and physical mechanisms (deterioration of ventilation in the root zone, which negatively affects the availability of oxygen, in addition to the removal of nutrients from the absorption range by the roots as a result of washing them away, which in turn affects the growth and productivity of the plant) [11,12]. Moreover, a clear decrease in the moisture content of sandy clay soils irrigated with low-salinity water compared to treatments irrigated with water of electrical conductivity of 3, 5 and 7 dS m^−1^ was noted [13,14]. The reason was attributed to the fact that plants growing in soils irrigated with low-salinity water are not exposed to high potential during water absorption. This indicates an increased ability of the plant to absorb irrigation water compared to plants growing in soils irrigated with water of high electrical conductivity. This is because water in the soil cannot be completely free, but is subject to a set of chemical and physical forces, and this is known as (water potential), which is a measure to determine the ability of water to move from one medium to another, and it represents a set of components, the most important of which are (matric potential and osmotic potential). In saline soils, the osmotic potential becomes more negative and dominant, as it turns salinity into physiological water stress even when irrigation water is available in the soil and within the field capacity.

In all soils, humic acids are produced as a result of the decomposition of organic matter. These acids contribute to plant nutrition by increasing the availability of mineral elements in the soil and promoting root growth, which in turn increases nutrient absorption. Furthermore, they enhance enzyme activity and cell division. Also, to mitigate the effects of salinity, organic amendments such as humic acids can improve soil structure and nutrient availability [15]. Another study demonstrated the effect of varying concentrations of humic acid (0, 60, 90, and 120 mL per 100 L) combined with NPK chemical fertilization [16]. This showed that adding humic acid increased tomato yield, the number of fruits, and fruit size. Refs. [17,18] noted that adding organic fertilizer to the experimental soil at a rate of 10 and 20 Mg ha^−1^ led to a clear and significant increase in the number of fruits, leaf area, total plant yield, and dry weight when adding the 20 Mg ha^−1^ level of organic fertilizer. Due to the worsening water scarcity problem in Iraq in general and in the south in particular, and the lack of research conducted to increase tomato production, this research was carried out with the aims of 1: Analyzing of the hydrological response and productivity of the tomato crop, as well as the physical response of the soil when using irrigation water of different quality and in the presence of varying levels of soil amendments, by measuring indicators of irrigation water use efficiency, water productivity, tomato crop, mean weight diameter, and saturated water conductivity, to establish constants and scientific foundations for water management and increase soil productivity; 2: Investigating the appropriate and optimal interactions between experimental factors (water quality and soil improvers) in a way that contributes to reducing dependence on low-salinity water without causing negative effects and a significant impact on crop productivity and also supports rationalization as a result of optimal water use.

## 2. Results

The field experiment was conducted on a tomato crop over two agricultural growing seasons. Two different irrigation water quality levels with varying electrical conductivity and three levels of organic fertilizers were used. According to the multivariate test, the study result found that the growing year did not have a significant effect on some of the studied characteristics due to the lack of clear variation in climatic factors in the experimental field environment. Significance was limited to the saturated hydraulic conductivity (Ks) and Mean Weight Diameter (MWD) only, while irrigation water quality was the most important and a highly significant factor, as were organic amendments (Table 1). The ratio of water quality to organic amendments was highly significant for some of the studied traits.

### 2.1. Water Use Efficiency

**Figure 1:**
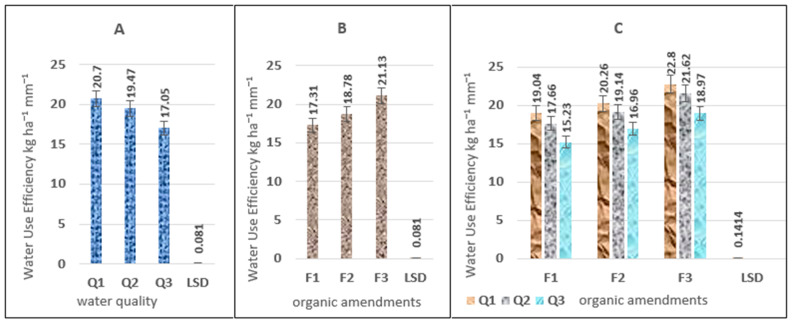
The effect of irrigation water salinity (**A**), soil improvers (**B**), and the interaction between them (**C**) on water use efficiency (WUE) values (kg h^−1^ mm^−1^).

The results of the statistical analysis of the F-test (Table 1) show a highly significant effect of both irrigation water salinity rotation and organic amendments, and their interaction, on WUE values. However, the study year and its interactions did not show any significant effect on the studied trait. The study results showed an increase in water use efficiency (WUE) averaging 20.7 and 21.13 kg ha^−1^ mm^−1^ when using freshwater and a high level of organic matter compared to the other treatments (water quality and soil amendments), respectively. The Q1 treatment achieved the highest WUE values at 20.7 kg ha^−1^ mm^−1^, followed by treatment Q2 at 19.47 kg ha^−1^ mm^−1^, while the lowest values, 17.05 kg ha^−1^ mm^−1^, were recorded in treatment Q3 (Figure 1A). Regarding the change in WUE values with organic amendments, the results in Figure 1B show a significant advantage for treatment F3, yielding the highest values compared to treatments F2 and F1. The average values for all treatments were 21.13, 18.78, and 17.31 kg ha^−1^ mm^−1^, respectively. The results (Figure 1C) show a highly significant interaction effect between saltwater rotation and organic amendments on irrigation WUE as shown in Table S1 (Supplementary Materials). The highest significant differences were observed between the saltwater rotation treatment Q1 compared to Q2 and Q3 for all organic amendments.

### 2.2. Water Productivity

**Figure 2:**
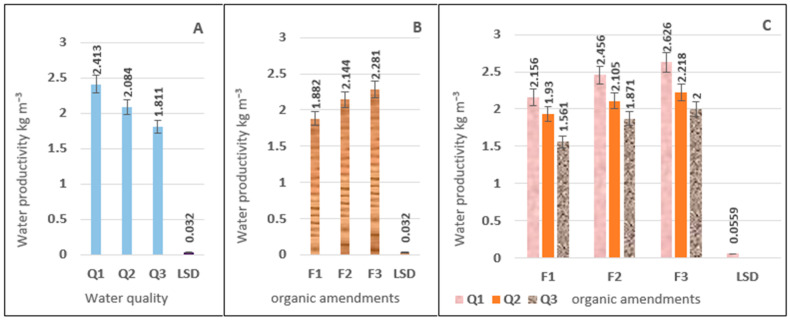
The effect of irrigation water salinity (**A**), soil improvers (**B**), and the interaction between them (**C**) on water productivity (WP) values (kg m^−3^).

Table 1 illustrates the effect of the study treatments on water productivity (WP) values. The table shows a significant interaction between irrigation water salinity, rotation and organic amendments on WP values. The WP values varied according to irrigation water salinity, reaching their highest values at Q1 compared to Q2 and Q3 for all soil improvement treatments. The highest values recorded with the interaction of organic amendments (F1, F2, and F3) were 2.156, 2.456, and 2.626 kg m^−3^ for rotation type Q1 compared to Q2 and Q3, respectively. It is noted from the results that the values of WP decrease for the salinity rotation Q3 overlapping with soil improvers (F1, F2, and F3) compared to Q2 and Q1 overlapping with the same soil improvers, and the percentages of decrease were (9.82, 23.83)%, (11.11, 23.81)% and (19.11, 27.59)% respectively (Figure 2C). Figure 2A shows a clear advantage in WP for treatment Q1, which recorded 2.413 kg m^−3^, whereas treatments Q2 and Q3 gave the lowest values, with an overall average of 2.084 and 1.811 kg m^−3^, respectively, representing an increase of 15.78% and 33.24% for the two transactions (Q2, Q3), respectively. Figure 2B shows that the soil improvement treatment affects WP values, with different treatment regimes. Treatment F3 outperformed the other soil correction treatments under study, recording the highest value of 2.281 kg m^−3^, while the values for treatments F2 and F1 were 2.144 and 1.882 kg m^−3^, respectively as shown in Table S2 (Supplementary Materials).

### 2.3. Mean Weighted Diameter of Water-Stable Soil Aggregates

**Figure 3:**
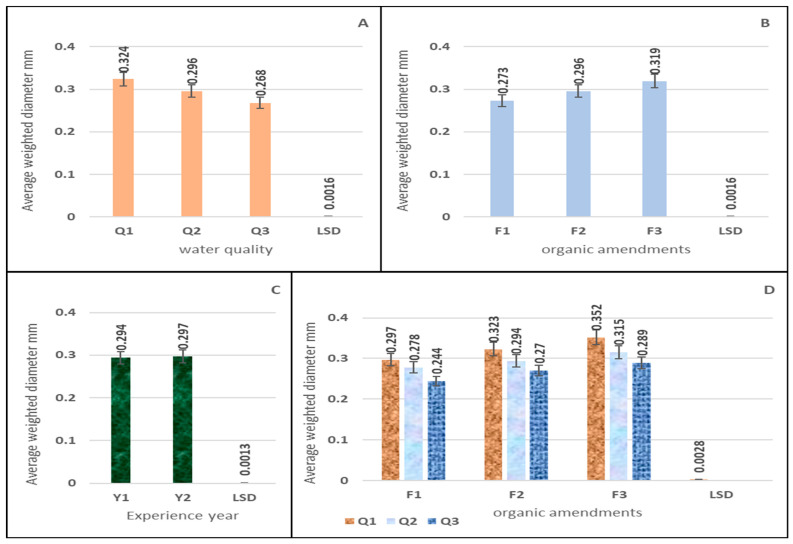
The effect of irrigation water salinity (**A**), soil improvers (**B**), experience year (**C**), and the interaction between them (**D**) on the values of the mean weighted diameter (MWD) (mm).

The results of ANOVA Table 1 show that there is a significant effect of both experimental factors, namely the irrigation water salinity rotation and the soil amendment additives, and their interaction on the weighted diameter average values. The Q1 rotation level gave the highest value at 0.324 mm, treatment Q2 recorded 0.296 mm, while the lowest value was 0.268 mm in treatment Q3 (Figure 3A). If the values of MWD change with soil improvers, the results in (Figure 3B) indicate that there is a clear significant superiority for treatment F3, which gave the highest values compared to F2 and F1, as the average values for all treatments were recorded as 0.319, 0.296, and 0.273 mm, respectively. Table 1 shows a significant effect of the year of the trial on the MWD values in mm. Figure 3C shows the significant differences in comparisons. This indicates a limited practical effect, which in turn is likely to be the result of a minor structural or biological response in the soil, rather than a fundamental change in its physical properties. Treatment Y2 recorded the highest values at 0.297 mm, while treatment Y1 yielded the lowest values at 0.294 mm. The results (Figure 3D) showed a significant effect resulting from the interaction between irrigation water rotation and organic amendments on MWD values as shown in Table S3 (Supplementary Materials). It was found that the highest significant differences were between irrigation rotation treatment Q1 compared to Q2 and Q3 for all organic amendments.

### 2.4. Saturated Hydraulic Conductivity

**Figure 4:**
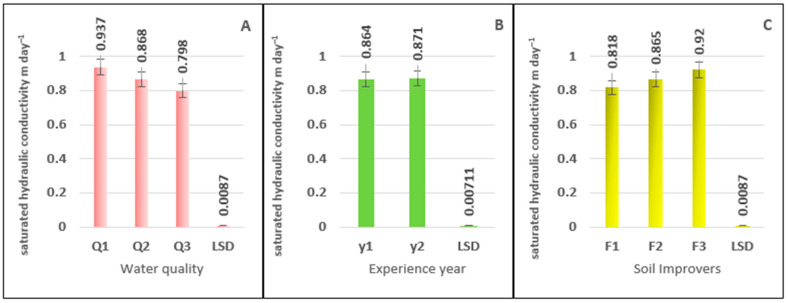
The effect of irrigation water salinity (**A**), experience year (**B**), and soil improvers (**C**), on saturated hydraulic conductivity (Ks) values (m day^−1^).

The results of the calculated F-test (1) statistical analysis show a highly significant effect of water quality on Ks values. Comparing the treatments revealed significant differences (Figure 4A), with treatment Q1 showing a significant increase in Ks values of 7.94% and 17.41%, respectively, compared to treatments Q2 and Q3, which had values of 0.937 m day^−1^. The variation in Ks values according to the experimental year is shown in Table 1, which indicates significant differences in these values. Figure 4B clearly shows significant differences between the study years. The Ks values decreased in the first year, recording 0.864 m day^−1^, while they increased to 0.871 m day^−1^ in the second year. Figure 4C shows a significant effect of Ks values at all levels of organic fertilizer application. Treatment F3 achieved the highest values at 0.920 m day^−1^, while F1 and F2 recorded the lowest values at 0.865 and 0.818 m day^−1^, respectively, as shown in Table S4 (Supplementary Material).

### 2.5. Yield

**Figure 5:**
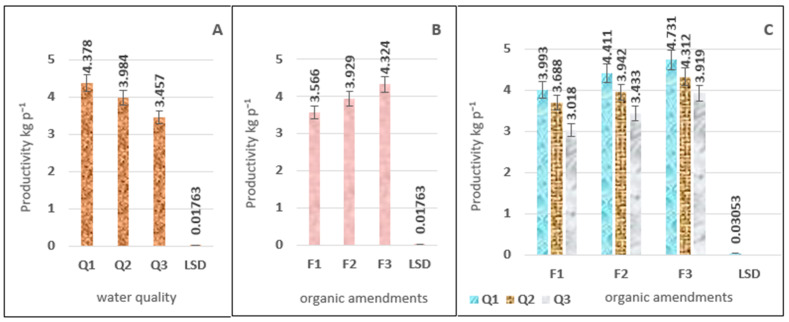
The effect of irrigation water salinity (**A**), soil improvers (**B**), and the interaction between them (**C**) on yield values (kg plant^−1^).

Table 1 (ANOVA) shows the significant effects of both main factors, namely the quality of irrigation water and organic amendments, as well as their interaction, on yield values. Rotation Q1 recorded the highest values at 4.378 kg plant^−1^, followed by treatment Q2, which gave 3.984 kg plant^−1^, while the lowest values, at 3.457 kg plant^−1^, were in treatment Q3, the percentage increase in the tomato yield for (Q1) compared to (Q2) and (Q3) was 0.093 and 0.266, respectively. Meanwhile, the percentage increase for (Q2) compared to (Q3) was 0.152 (Figure 5A). Regarding the variation in yield values with organic fertilizer levels, the results in Figure 5B show a significant advantage for treatment F3, which recorded the highest values compared to treatments F2 and F1. The average values for all treatments were 4.324, 3.929, and 3.566 kg plant^−1^, respectively. The results in Figure 5C show a highly significant interaction effect between the two field experiment factors on yield values as shown in Table S5 (Supplementary Materials). The highest significant variations were observed between irrigation water quality treatment Q1 compared to Q2 and Q3, and across all organic fertilizer application levels. The water quality treatment Q2 with soil amendment F3 achieved the highest tomato yields compared to the freshwater treatment Q1 with soil amendment F1, reaching 4.312 and 3.993 kg per plant, respectively.

## 3. Discussion

The reason for the increased WUE value of treatment Q1 compared to the other treatments (Q2 and Q3) is due to the increased vegetative growth of tomatoes and thus an increase in their productivity per unit area. This comes from the use of water with a low salt content, which increases the plant’s ability to improve the absorption of irrigation water and nutrients due to the higher (less negative) osmotic potential (ψs), which in turn improves metabolic efficiency. Also, the use of low-salinity irrigation water reduces the need for additional water requirements for soil leaching. The superiority of treatment F3 over the other treatments may be attributed to the fact that increased organic fertilizer application improves soil structure, leading to increased water-holding capacity (WHC) and reduced water loss through evaporation and surface runoff [18]. This, in turn, reduces the need for frequent and close irrigation. Furthermore, it increases the activity of soil microorganisms, which results in nutrient mineralization, making them more available to plants. Organic fertilizers also reduce water stress on plants by minimizing water loss through irrigation. All of these factors contribute to better vegetative growth and higher tomato yields [19]. The reduced irrigation water use efficiency resulting from drainage water rotation Q2 and Q3 leads to increased water loss without achieving balanced vegetative growth, which in turn reduces plant productivity and the plant’s ability to utilize available nutrients [13].

The decrease in Ks values when using irrigation water with relatively high salinity may be attributed to the physical effects on soil porosity and structure, which are represented by the dispersion of clay particles, and thus a decrease in the absorption of mineral nutrients by the plant. Also, the osmotic pressure in the soil increases as a result of using saline water, which makes it difficult for the plant to absorb irrigation water, even if it is available in the soil [20]. The increased water productivity in low-salinity water is attributed to improved soil fertility, specifically its structure and increased water and air permeability, which enhances the plant roots’ ability to absorb nutrients and water. Moreover, it increases the activity of soil microorganisms, which provide essential nutrients to the plant, leading to higher yields [8]. The increased water productivity of treatment F3 is attributed to the improvement in the soil’s physical and chemical properties, which enhanced the soil’s ability to retain water through decreased evaporation and deep percolation, thus reducing the frequency of irrigation. Furthermore, the increased organic matter in the soil creates a more stable environment for plants, mitigating the effects of drought and increasing irrigation water use efficiency [21].

The increase in the MWD value for treatment Q1 compared to the other treatments (Q2 and Q3) is attributed to the decrease in salinity in the irrigation water, which in turn improves the activity of microorganisms resulting from the decrease in osmotic pressure in the soil [13]. Also, the stability of soil aggregates increases and their dispersal is reduced as a result of the abundance of calcium (Ca^2+^) in the soil solution during irrigation in water with a low salinity level. While the MWD readings decrease with increasing salinity of irrigation water, and increasing the exchangeable sodium ratio (SAR) displaces magnesium and calcium from cation exchange sites, which in turn leads to increased disintegration of soil aggregates and also reduces biological activity in the soil [14]. The superiority of treatment F3 over other treatments may be attributed to the fact that increasing the amount of organic fertilizer added increases the activity of the microorganisms, as organic fertilizer is a rich source of energy (organic carbon) for these microorganisms. Soil structure also improves because organic fertilizer contains sticky and binding substances such as organic acids, which act as an adhesive that binds soil particles together. Organic matter also increases the soil’s nutrient retention and cation exchange capacity (CEC), which in turn contributes to the presence of cations that increase soil structural integrity [22]. The higher MWD values in soil Y2 compared to soil Y1 are attributed to the increased organic matter resulting from the decomposition of plant roots and other crop residues, which acts as a binding agent for soil particles. Additionally, improved moisture distribution and aeration due to repeated planting, which creates pores and channels in the soil body from older roots, also contribute to the higher MWD values. The decrease in MWD values resulting from the interaction between salinity rotation Q2 and Q3 and organic fertilizer treatments is attributed to the effect of salts in irrigation water on soil structure, even in the presence of organic fertilizer. Sodium leads to the dispersion of clay particles, resulting in the collapse of soil structure. Organic fertilizer requires (low salinity and moderate moisture) to activate biological activity in the soil, thus reducing the decomposition of organic matter and affecting the soil’s ability to form stable aggregates [23].

The treatment Q1 showed a significant increase in Ks values compared with Q2 and Q3. This is attributed to the role of low-salinity water in maintaining soil porosity and minimizing salt deposition. This, in turn, allows water to move easily and smoothly within the soil profile, unlike irrigation with saline water, which degrades soil permeability due to salt deposition [24]. The superiority of the second year is attributed to the continuity of soil cultivation, which resulted in an improved soil structure. This improvement resulted from root-mediated macro porosity in the second year, which increased water movement and, consequently, soil conductivity. Additionally, the plant residues remaining from the previous season played a vital role in improving soil aeration and water retention, along with the frequent irrigation and the resulting leaching of salts from the soil. The superiority of treatment F3 compared to treatments F1 and F2 is attributed to the increased amount of organic matter added, which modified and improved the soil’s physical properties, including density. This, in turn, positively affected porosity and soil structure, making the soil more permeable and capable of water transmission [25].

The reason for the increased productivity of treatment Q1 compared to the other study treatments (Q2 and Q3) is due to the increased promotion of flower formation and thus normal fruit set compared to saline water, which causes blossom-end rot (BER) and flower drop. Also, fresh water increases carbohydrate production and vegetative and fruit plant growth as a result of improving photosynthetic efficiency. This shows that the use of low-salinity irrigation water can reduce the effects of salt stress, represented by (nutrient malabsorption and ion toxicity) [26]. The superiority of treatment F3 over the other experimental treatments may be attributed to the fact that increasing the amount of added organic fertilizer stimulates beneficial microorganisms that fix nitrogen and nutrient mineralization. It also stimulates the secretion of growth regulators, which in turn promote vegetative growth in tomatoes. Furthermore, it provides macro and micronutrients after decomposition, which support the flowering and fruiting of the economically important plant. In addition, it improves the physical properties of the soil, such as density and porosity, leading to easier water movement and improved root aeration, thus providing a suitable environment for root growth and increasing its absorption capacity [27]. The decrease in productivity resulting from the two salinity rotation treatments Q2 and Q3 was attributed to ionic stress and osmotic stress. These are the two-phase growth response to salinity caused by the accumulation of chloride and sodium ions, leading to plant cell toxicity. Additionally, osmotic stress reduced water uptake by tomato roots, and a nutrient imbalance occurred, as high sodium levels competed with and affected potassium, while high chloride levels reduced nitrate uptake. All of these factors resulted in impaired vegetative growth and incomplete fruit set, leading to the production of small, poor-quality fruits [14,28].

## 4. Materials and Methods

### 4.1. Description of Experimental Location, Climate, and Soil

**Figure 6:**
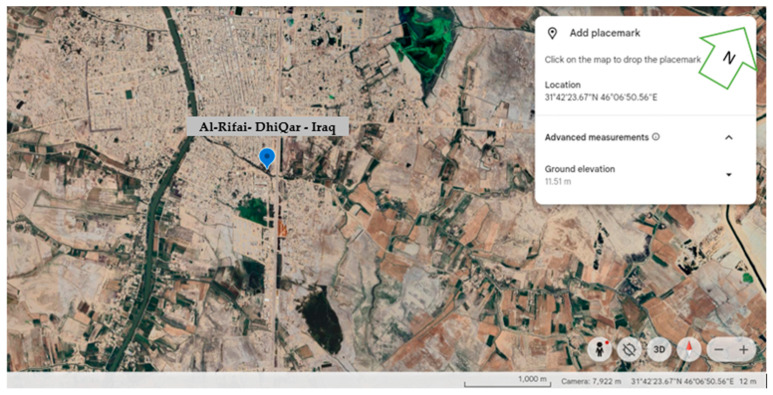
Experimental site location.

The experiment was conducted during the autumn seasons of 2022–2023 and 2023–2024 in a greenhouse field near Al-Rifai District, located in Dhi Qar Governorate (Nasiriyah, Iraq). The soil has a silty clay loam texture. It is located at latitude 31°43′20″ N and longitude 46°6′30″ E (Figure 6). The governorate is located in southern Iraq, north of Basra Governorate.

The field experiment site is located within the almost arid climatic sector, which characterizes southern Iraq. The main climatic factors that were prevalent during the period of implementation of the applied experiment have been included in Table 2.

The soil used in the study had a silty clay loam texture. The surface of the experimental soil was leveled inside the greenhouses after plowing and smoothing [29]. Soil samples were taken after the preparation processes were completed to a depth of 0–30 cm, and plant remains were removed from the soil samples. It was then air-dried, ground well, and passed through a 2 mm diameter sieve to obtain some chemical and physical properties [30,31]. The main properties of the soil are summarized in Table 3 (as averages for both growing seasons).

### 4.2. Plant Materials

The seeds were selected from the Super Strain B Tomato Cultivar and then planted in cork trays containing suitable soil prepared for this purpose, at a rate of one seed per location, after which the necessary service was carried out.

### 4.3. Experimental Procedure

**Figure 7:**
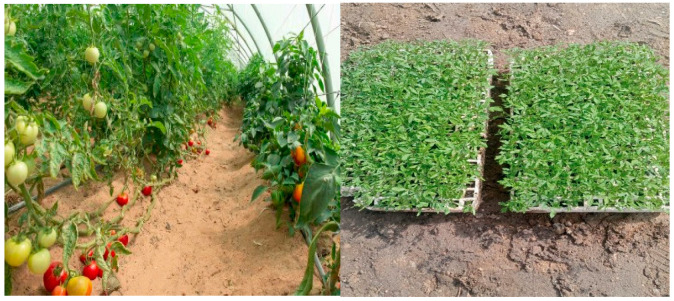
**Right** images represent the field experiment of tomato cultivation in the canals, **Left** image represent tomato seedlings before planting of Al-Rifai city, in Dhi Qar Governorate Iraq.

The experiment was designed using the system of Randomized Complete Block Design (RCBD), with three replicates. The treatments, which numbered 9, were randomly distributed in each block separately, so that the total number of experimental units became 27 experimental units distributed over three plastic greenhouses. The greenhouse size was 22 × 9 m as shown in (Figure 7).

### 4.4. Experimental Parameters and Studied Characteristics

Water quality, A: (Q1) that is, the plants were irrigated throughout their life with water with a salinity of 0.8 dS m^−1^. B: (Q2) That is, the irrigation water is given with low salinity in the first irrigation, 0.8 dS m^−1^, and in the second irrigation, it is irrigated with water with high salinity, 5.8 dS m^−1^, and so on. The irrigation process is repeated; that is, in the third irrigation, it is irrigated with fresh water, while in the fourth irrigation, water with high salinity is used. C: (Q3) Here, saline water (5.8 dS m^−1^) is used for irrigation throughout the growing season.

Soil improvers: These are 0 Mg ha^−1^ (without additives, symbolized F1), 15 Mg ha^−1^ (symbolized F_2_), and 30 Mg ha^−1^ (symbolized F_3_). The experiment was conducted in three greenhouses. A drip irrigation system was designed with a spacing of 25 cm between emitters and 90 cm between adjacent rows. The length of the planting row was 20 m, and the distance between plants on the same row was 45 cm. (Cow manure was used as a soil conditioner after being fermented for one year. The nitrogen content was estimated at 1.1% of the dry weight. The phosphorus content was 0.5% of the dry weight, and the total potassium content was 0.7% of the dry weight, while the carbon-to-nitrogen ratio was 13:1.)

The following indicators were studied in this research:

Water Use Efficiency (WUE)

The efficiency was calculated for each independent experimental unit based on both the dry weight of the vegetative mass and the amount of irrigation water consumed by the plant, using the mathematical formula provided in [32]:

(1) $$WUE=\frac{Ya}{Water\;applied}$$

where

WUE: Water use efficiency (kg ha^−1^ mm^−1^), Ya: crop production (kg plant^−1^), Water applied: Depth of added water (mm).

Water Productivity (WP)

Water productivity refers to the relationship between production per unit volume of water added in the irrigation process and is expressed according to the formula given in [33]:

(2) $$(WP)=\frac{Yield}{Vw}$$

where:

WP: Water productivity (Kg m^−3^), Yield: Productivity (Mg ha^−1^), Vw: Volume of water added (m^3^ ha^−1^).

Mean Weight Diameter (MWD)

It was measured using the well-known wet sieving method, and the results were expressed as the MWD by applying the equation described by [34] and mentioned by [35], as follows:

(3) $$MWD=\sum_{i=1}^{n}XiWi$$

where:

MWD: Weighted average diameter (mm), Xi: Average diameter for any size range of separated aggregates (mm), Wi: Weight of remaining aggregates within a single size range as a percentage of the total dry weight of the soil sample.

Saturated Hydraulic Conductivity (Ks)

Conductivity was measured by taking a sample of the experimental soil and following the constant-head method proposed and described in [35]. The values of saturated hydraulic conductivity of the soil were calculated by applying Darcy’s well-known law as follows:

(4) $$Ks=\frac{V}{At}\times \frac{L}{∆H}$$

where:

Ks: Saturated water conductivity (m day^−1^), V: Volume of water passing through the soil column (cm^−3^), A: Cross-sectional area of the soil column (cm^2^), t: Time (min), L: Length of the soil column (cm) + H: Height of water column above the soil column (cm).

Average yield per plant (kg)

This characteristic is important as it shows the effect of experimental factors and their interactions on the results obtained from production. It is calculated by dividing the total yield for each greenhouse by the number of plants growing within it.

### 4.5. Statistical Analysis

The field experiment data were statistically analyzed using Genstat version 10.30E (2010). All studied physical and yield characteristics were analyzed according to the experimental design adopted for this study. Analysis of variance (ANOVA) was performed to determine the significance of differences between the coefficients of the various factors at established statistical significance levels. When statistically significant differences were found, the least significant difference (LSD) (*p* < 0.05) test was used to determine the direction of the differences between the treatments. The final results were presented as statistical means supported by statistically significant values to facilitate the interpretation of the effect of the different treatments [36].

## 5. Conclusions

This study addressed the challenge of water scarcity and improving water utilization strategies to enhance irrigation efficiency and increase production under dry climatic conditions resulting from climate change and global warming in the region. The rotation method resulted in a 50% reduction in fresh water consumption, which is crucial as it provides a viable solution to the problem of fresh water scarcity in the country in general, and in the south in particular. The Q2 rotation is a viable alternative that maintains acceptable productivity levels. Exploring alternatives to low-salinity irrigation water is one of the objective solutions to the problem of scarcity of fresh surface water in Iraq. Thus, drainage water or even moderately saline groundwater can be utilized, which was used in irrigation in conjunction with specific levels of organic fertilizer, which in turn improved the studied characteristics and increased tomato yield. The research focused on identifying irrigation strategies with saline water and their effect on some physical soil properties, and evaluating the effect of organic fertilizer addition rates on yield, whether with fresh water irrigation or rotation. The study results confirm the feasibility and short-term sustainability of using saline water in rotation with fresh water, combined with organic fertilizer, to achieve optimal irrigation efficiency and tomato production. The results showed that rotation irrigation with organic fertilizer significantly impacted the studied characteristics and tomato yield. Specialized researchers can test this irrigation system in the sandy soils of southern Iraq, where physical properties, including water retention, differing hydraulic properties and matric potentials. Studies should also be conducted to investigate the effects of saline irrigation water on soil microbial health and mineral availability. To achieve optimal results, tomato farmers in southern Iraq should adopt the Q2 irrigation cycle, which provides a balance between water use efficiency, tomato yield, and certain soil physical properties. This study suggests adopting a strategy of rotating saline water (or water within a specific salinity range) with freshwater, combined with the application of organic amendments.

## Figures and Tables

**Table 1:** Shows the analysis of variance for the tabular F values for the studied traits.

Source	df	Water Use Efficiency (kg ha^−1^ mm^−1^)	Water Productivity (Kg m^−3^)	Mean Weight Diameter of Water-Stable Soil Aggregates (mm)	Saturated Hydraulic Conductivity (m day^−1^)	Average Yield per Plant (kg)
	1	NS	NS	18.89 **	3.99 *	NS
Q	2	4250.55 **	716.78 **	2366.66 **	523.21 **	5659.87 **
F	2	4573.49 **	323.75 **	1593.02 **	284.40 **	3800.92 **
Y + Q	4	NS	NS	NS	NS	NS
Y + F	4	NS	NS	NS	NS	NS
Q × F	5	11.27 **	6.98 **	24.88 **	NS	49.75 **
Y + Q + F	7	NS	NS	NS	NS	NS

Y: -Study year, F: -Soil Improvers, Q: -rotation method, df: Degrees of freedom, ** Significant at 0.01 level. * Significant at 0.05 level, NS: no significant.

**Table 2:** Shows the meteorological data for the experimental site in the two growing seasons (2022/2023 and 2023/2024).

Autumn Tomato Planting Season 2022/2023						
	Oct.	Nov.	Dec.	Jan.	Feb.	Mar.
Precipitation (mm)						
	1.53	1.03	2.42	0.64	1.76	1.95
Temperature (°C)						
Max.	35	25.8	15.8	14.1	17.41	24.36
Min.	19.7	17.3	5.8	3.5	2.3	10.22
Average	27.35	21.55	10.8	8.8	9.85	17.29
The total monthly solar radiation (W m^−2^)						
	759	623	377.2	164	151.83	476.3
Autumn tomato planting season 2023/2024						
	Oct.	Nov.	Dec.	Jan.	Feb.	Mar.
Precipitation (mm)						
	0.53	1.205	3.37	1.05	0.92	1.04
Temperature (°C)						
WeMax.	35.5	26.2	16.1	14.8	19.41	25.85
Min.	18.3	17.9	6.1	3.9	4.4	10.8
Average	26.9	22.05	11.1	9.35	11.9	18.32
The total monthly solar radiation (W m^−2^)						
	714.3	673.12	402.1	234.45	161.05	422.03

**Table 3:** Shows some properties of the physical study soil before planting.

Properties	Soil Depth (0–30 cm)
BD (g cm^−3^)	1.282
Mean Weight Diameter (MWD) (mm)	0.301
Sand (g kg^−1^ soil)	156
Silt (g kg^−1^ soil)	477
Clay (g kg^−1^ soil)	367
Soil texture	Silty clay loam
pH	7.43
EC ds m^−1^	1.9
Porosity %	47.9
PD (g cm^−3^)	2.55

Soil properties analyzed in the laboratory of University of Summer, Al-Rifai District, located in Dhi Qar Governorate (Nasiriyah, Iraq).

## Data Availability

The original contributions presented in this study are included in the article and Supplementary Material. Further inquiries can be directed to the corresponding author.

## Supplementary Materials

The following supporting information can be downloaded at: https://www.mdpi.com/article/10.3390/plants15050734/s1, Table S1: Effect of the interaction between rotation salinity and soil improvers on water use efficiency (WUE) values (kg h^−1^ mm^−1^); Table S2: Effect of the interaction between rotation salinity and soil improvers on water productivity (WP) values (kg m^−3^); Table S3: Effect of the interaction between rotation salinity and soil improvers on the values of the mean weighted diameter (MWD) (mm); Table S4: Effect of the interaction between rotation salinity and soil improvers on saturated hydraulic conductivity (Ks) values (m day^−1^); Table S5: Effect of the interaction between rotation salinity and soil improvers on yield values (kg plant^−1^).
