# Supplementary material for: Water Use Efficiency and Tomato Yield Under the Influence of Irrigation Water Quality and Soil Improvers Using a Drip Irrigation System
Source: Plants (Basel). 2026 Feb 28;15(5):734. doi: 10.3390/plants15050734 (PMC12986883; doi:10.3390/plants15050734)
Supplement: Supplementary file 1 [file plants-15-00734-s001.zip › plants-4144890-supplementary.pdf]

Supplementary Materials

Table S1. Effect of the interaction between rotation salinity and soil improvers on water use efficiency (WUE) values (kg h^{-1} mm^{-1}).

Water Use Efficiency (WUE)				
Water Quality	Interaction between water quality and soil improvers			Average water quality
	F1	F2	F3	
Q1	19.04	20.26	22.8	20.7
Q2	17.66	19.14	21.62	19.47
Q3	15.23	16.96	18.97	17.05
LSD ETIWS		0.1414		LSD wq 0.081
Average Soil Improvers	17.31	18.78	21.13	
		0.081		

Table S2. Effect of the interaction between rotation salinity and soil improvers on water productivity (WP) values (kg m^{-3}).

water productivity (WP)				
Water Quality	Interaction between water quality and soil improvers			Average water quality
	F1	F2	F3	
Q1	2.156	2.456	2.626	2.413
Q2	1.93	2.105	2.218	2.084
Q3	1.561	1.871	2	1.811
LSD ETIWS		0.0559		LSD wq 0.032
Average Soil Improvers	1.882	2.144	2.281	
		0.032		

Table S3. Effect of the interaction between rotation salinity and soil improvers on the values of the mean weighted diameter (MWD) (mm).

mean weighted diameter (MWD)				
Water Quality	Interaction between water quality and soil improvers			Average water quality
	F1	F2	F3	
Q1	0.297	0.323	0.352	0.324
Q2	0.278	0.294	0.315	0.296
Q3	0.244	0.27	0.289	0.268
LSD ETIWS		0.0028		LSD wq 0.0016
Average Soil Improvers	0.273	0.296	0.319	
		0.0016		
Experience Year	Y1	Y2	LSD EY	
	0.294	0.297	0.0013	

Table S4. Effect of the interaction between rotation salinity and soil improvers on saturated hydraulic conductivity (Ks) values (m day^{-1}).

Saturated Hydraulic Conductivity (Ks)					
Average Water Quality	Values	Average Soil Improvers	Values	Average Experience Year	Values
Q1	0.937	F1	0.818	Y1	0.864
Q2	0.868	F2	0.865	Y2	0.871
Q3	0.798	F3	0.92		
LSD WQ 0.081	0.0087	LSD SI 0.081	0.0087	LSD AE 0.081	0.00711

Table S5. Effect of the interaction between rotation salinity and soil improvers on yield values (kg $plant^{-1}$).

Yield values (kg $plant^{-1}$)				
Water Quality	Interaction between water quality and soil improvers			Average water quality
	F1	F2	F3	
Q1	3.993	4.411	4.731	4.378
Q2	3.688	3.942	4.312	3.984
Q3	3.018	3.433	3.919	3.457
LSD ETIWS		0.03053		LSD wq 0.01763
Average Soil Improvers	3.566	3.929	4.324	
		0.01763		
